# Pediatric Migraine and Visual Cortical Excitability: A Prospective Observational Study with Sound-Induced Flash Illusions

**DOI:** 10.3390/children11040394

**Published:** 2024-03-26

**Authors:** Salvatore Di Marco, Laura Pilati, Angelo Torrente, Simona Maccora, Andrea Santangelo, Giuseppe Cosentino, Edvige Correnti, Vincenzo Raieli, Brigida Fierro, Filippo Brighina

**Affiliations:** 1 Department of Biomedicine, Neuroscience and advanced Diagnostics (BiND), University of Palermo, 90127 Palermo, Italy; dimarcosal@gmail.com (S.D.M.); laura.pilati.91@gmail.com (L.P.); angelo.torrente@unipa.it (A.T.); simona.maccora@unipa.it (S.M.); brigida.fierro@unipa.it (B.F.); filippo.brighina@unipa.it (F.B.); 2 Neurology and Stroke Unit, P.O. “S. Antonio Abate”, 91016 Trapani, Italy; 3 Neurology Unit, ARNAS Civico di Cristina and Benfratelli Hospitals, 90127 Palermo, Italy; 4 Pediatrics Department, AOUP Santa Chiara Hospital, 56126 Pisa, Italy; a.santangelo9@studenti.unipi.it; 5 Translational Neurophysiology Research Unit, IRCCS Mondino Foundation, 27100 Pavia, Italy; giuseppe.cosentino@unipv.it; 6 Department of Brain and Behavioral Sciences, University of Pavia, 27100 Pavia, Italy; 7 Child Neurology and Psychiatry Unit—ISMEP, “G. Di Cristina” Children’s Hospital—ARNAS Civico, 90127 Palermo, Italy; edvige.correnti@arnascivico.it

**Keywords:** pediatric migraine, cortical excitability, sound-induced flash illusion, headache

## Abstract

The pathophysiological mechanisms underlying migraine are more difficult to investigate in children than in the adult population. Abnormal cortical excitability turns out to be one of the most peculiar aspects of migraine, accounting for the manifestations of migraine attacks. Recently, visual cortical excitability has been explored effectively in adult migraineurs with a technique based on cross-modal audio-visual illusions (with sound-induced flash illusions (SIFIs) being reduced in migraineurs compared to non-migraineur subjects). On such a basis, in this study, we investigated visual cortical excitability in children with migraine using SIFIs using combinations of visual and sound stimuli presented randomly. We evaluated 26 children with migraine without aura and 16 healthy children. Migraineurs did not differ from the age-matched healthy subjects regarding fission or fusion illusions but perceived more flashes in trials of multiple flashes with or without beeps. The higher number of SIFIs in migraineur children compared to adults may be due to a greater propensity of visual stimulation to be driven by auditory stimuli (i.e., acoustic dominance). The increased ability to perceive flashes reveals a hyperfunctional visual cortex, demonstrating that the use of SIFIs is a valid tool for assessing visual cortical responsiveness even in pediatric migraine.

## 1. Introduction

Migraine is one of the most common forms of primary headache occurring during childhood [1]. About 1 out of 10 children suffer from migraine, even in the population under 7 years of age [2,3]. Migraine is considered an episodic and familial disorder characterized by recurrent headache episodes, widely varying in frequency, duration, and intensity. Furthermore, in adults, it is often responsible for relevant disability and high social and economic costs, in terms of healthcare expenditure (i.e., direct costs), loss of working days, and reduced productivity (i.e., indirect costs) [4]. An Italian study that investigated pediatric headache populations over six months found direct costs per child with migraine of EUR 802.80; moreover, the total indirect cost due to headache in the whole pediatric population was EUR 1323.30, with an average indirect cost per patient of EUR 52.97 (even considering parental work productivity loss) [5]. In addition to the linked societal costs, migraine represents one of the most associated causes of absenteeism from school [6]. 

During a migraine attack, the pain is usually accompanied by other symptoms such as nausea, pallor, phonophobia, and photophobia; moreover, in around a quarter of migraine patients, pain episodes are preceded or accompanied by transient focal neurologic symptoms, known as aura. Despite migraine usually being thought of as a disorder characterized by phasic transient cephalic pain episodes, the current knowledge is that it follows a cyclic pattern, with different phases. This “migraine cycle” sees (i) a pre-ictal (or prodromal) phase that may last up to 48 h before the attack, during which the patient experiences non-specific symptoms such as yawning, mood changes, or food craving; (ii) ictal phase represented by the headache and its associated symptoms; (iii) a post-ictal (or post-dromal) phase that may last up to 48 h and include other non-specific symptoms such as tiredness and fluid retention; and (iiii) an interictal phase between each post-dromal phase and the next pre-ictal phase [7,8,9]. Even though the interictal phase may be a headache-free time, several patients still experience some symptoms including subjective memory impairment, psychological symptoms, or constant photo- or phonophobia. 

In childhood, there are, in addition, some heterogeneous clinical signs related to migraines such as infant colic, abdominal migraine, cyclic vomiting, and benign paroxysmal vertigo, once considered possible precursors of the disease (i.e., migraine equivalent) [1,10]. Differently from the adult form, pediatric migraine is characterized by attacks of a shorter duration, less pronounced lateralization [11], and an aura that may be atypical [12], and it may be accompanied by cranial autonomic disturbances [13]. In addition, migraineur children may present a different response to preventive drugs from adults [2]. 

The human brain shows a maturation that extends over time, and the developmental trajectories are different in different brain structures, neural circuits, and white matter. These dynamic changes are particularly relevant during the developmental age. For instance, longitudinal MRI studies in healthy children have shown progressive increases in white matter, reversed trajectories of grey matter, and increased connectivity in adolescence, while in developmental pathologies, changes in these typical trajectories can be observed [14,15]. For these and various other reasons, the child cannot be considered a small adult, especially from a neurological point of view [16].

Despite the large body of literature focused on this area, migraine etiopathogenesis has not yet been fully understood, but consistent evidence emphasizes the role of abnormalities of cortical excitability [17,18]. There are forms of migraine (familial hemiplegic migraine—FHM) in which the pathogenetic mutation depends on genes of some particular channel proteins involved in normal neuronal functioning and the control of cortical excitability [19,20]. Knock-in mice for these mutated genes showed a greater susceptibility to developing cortical spreading depression (CSD), a phenomenon considered to be the basis of migraine aura [21].

An important contribution to the evaluation of cortical abnormalities in migraine comes from studies based on neurophysiological investigations such as evoked potentials and non-invasive brain stimulation (NIBS) techniques like transcranial magnetic stimulation (TMS) and transcranial direct current stimulation (tDCS). These techniques revealed abnormal cortical excitability in migraine, particularly in the visual cortex [21,22]. Such results, suggesting that migraineurs show increased cortical excitability, have been supported both by studies with repetitive TMS (rTMS) and with a paired-pulse paradigm [23]. Even the phosphene threshold (PT, i.e., the lowest TMS intensity to induce the perception of phosphenes in a subject) was found altered in migraineurs. In basal conditions, they showed a reduced PT (reflecting an increased cortical excitability). Nevertheless, after an inhibitory low-frequency rTMS protocol on the occipital cortex, migraineurs seemed to have a paradoxical facilitatory effect, in contrast to the inhibitory effect on controls [24].

The excitability linked to the activation level of the occipital cortex plays an important role in the mechanisms of visual perception and the neural processes underlying cross-modal perception (i.e., the increased signal resulting from the interaction between two different sensory modalities). This phenomenon can be investigated by evaluating specifically induced illusory phenomena. One of the most used models is represented by sound-induced flash illusions (SIFIs), elaborated by Shams et al. to evaluate the audio-visual interaction of visual stimuli presented concurrently with auditory ones [25]. With this technique, when a single flash is accompanied by two or more beeps, the subject perceives more flashes than real ones (fission illusions); differently, fusion illusions arise when multiple flashes are presented with a single beep and are perceived as less than their real number. Even if the precise mechanisms underlying SIFIs remain to be defined, a critical role seems to be played by visual cortical excitability [26]. In 2011, Bolognini et al. [26] showed that artificially increasing the excitability of the occipital cortex through anodic facilitatory tDCS reduces the illusory phenomena. Considering the role of alterations in visual cortical excitability in migraine, Brighina et al. [27] evaluated the development of auditory-visual illusions in a group of adult migraine patients with and without aura, both in the interictal and in the ictal phases, and comparing data with healthy subjects. The results showed that in the case of fission illusion, all the subpopulations of migraineurs showed fewer illusions than controls. In this study, the cross-modal illusions were therefore demonstrated to be a valuable tool for exploring the functional connectivity between the sensory areas, which probably plays an important role in migraine pathophysiology [27].

During childhood, despite multisensory abilities already being present, as demonstrated by Innes-Brown et al. [28], the audio-visual illusory phenomenon is significantly increased compared to adults. This suggests that the selective integration ability of bimodal stimuli requires a very long period to develop completely (until late adolescence) [28]. Nava et al. [29] showed that the number of fission illusions presents a reduction trend with increasing age. This result may be correlated to the progressive maturation of multisensory integration systems during development, which sees the transition from an auditory dominance to a progressive visual one at an evolutionary age, implicating more and more emphasis on visual stimulation [29].

In the present study, the primary aim is to use SIFIs to evaluate the perception of the illusory auditory-visual phenomena in migraineur children compared with healthy controls in the same age range, to understand the role of abnormal excitability as related to basic pathophysiological mechanisms or as a marker of disease progression. Indeed, we hypothesized that as compared to healthy children, those affected by migraine would show a reduced extent of illusions, which means a condition of cortical hyperexcitability, as is found in adult migraineurs [30].

## 2. Materials and Methods

### 2.1. Participants

We enrolled pediatric subjects with migraine without aura diagnosed following the International Classification of Headache Disorders, third edition beta version (ICHD-3 beta) [31] criteria, with appropriate age-related variations. As inclusion criteria, children should have shown unremarkable neurological examination, no other comorbidities, normal or corrected vision by using graduated lenses, and normal hearing and should not have been undergoing any chronic or continuous pharmacological treatment. We also enrolled healthy subjects with the same age and sex distribution. During the inclusion, we also paid attention to avoiding the presence or any family medical history of migraine (among healthy children) or other neurological and psychiatric disorders (among all the subjects), which would have represented exclusion criteria.

All patients were examined during the interictal phase of the migraine cycle (i.e., at least 48 h after the last attack), and we checked for the absence of any new attack in the 48 h after the test through a telephone call. We performed the study only in patients affected by migraine without aura. This is because our principal aim was to explore the changes in cortical excitability related to migraine itself independently using cortical abnormalities linked to the mechanisms underlying aura. Moreover, migraine with aura presents a minor prevalence compared to migraine without aura, and considering the difficulties of recruiting pediatric patients, this aspect could have further reduced the chances of obtaining adequate populations for comparison. 

The study was approved by the Palermo 1 Ethics Committee (Palermo, Italy, protocol no. 5/2015 of 13 May 2015), and the children underwent the test after both the parents received adequate information and signed a specific informed consent. The inclusion period was from June 2015 to March 2017. Migraine patients were recruited from the ones referring to the pediatric headache center of Di Cristina Hospital, while healthy subjects were in a class of catechism in Palermo, Italy.

### 2.2. Stimulation and Task

**Figure 1:**
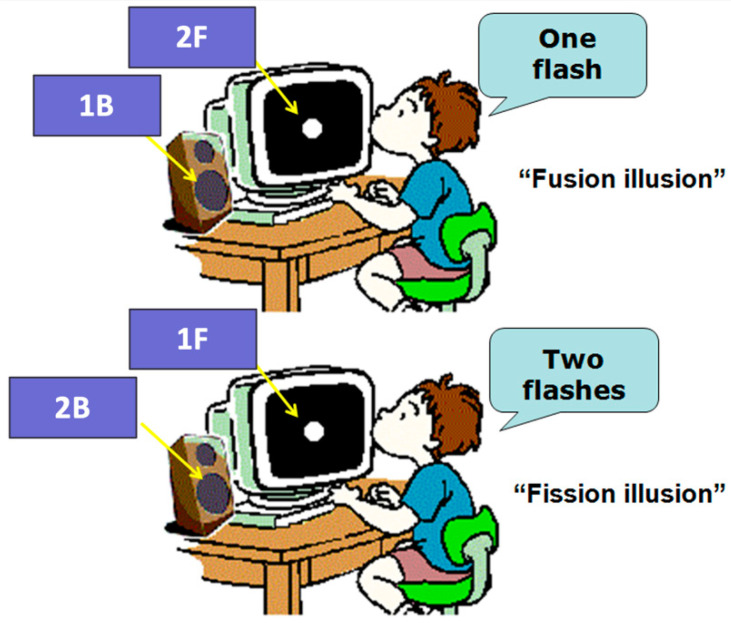
Simplification of the task required from the subjects.

The participants sat in a dark room in front of a black screen located about 70 cm distance from their eyes. We used E-prime software (version 2.0^®^, Psychology Software Tools, Pittsburgh, PA, USA) to present, in random order, visual stimuli as white flash disks and sound stimuli with the following characteristics: sound intensity of 95 dB, frequency of 3.5 kHz, duration of 7 msec, and administered 23 msec before the flash. We distinguished between single-flash trials and multiple-flash trials. In single-flash trials, the flash (F) was accompanied by 0 to 4 beeps (B) (i.e., 1F0B, 1F1B, 1F2B, 1F3B, 1F4B, where 1F2B, 1F3B, and 1F4B trials aimed to induce the fission illusions); in multiple-flash trials, 2 to 4 flashes were accompanied by 0 to 1 beep (i.e., 2F0B, 3F0B, 4F0B, 2F1B, 3F1B, 4F1B, where 2F1B, 3F1B, and 4F1B trials aimed to induce the fusion illusions). Thus, the total number of combinations was 11. The task of the subjects was to fix the center of the screen and judge the number of flashes seen during each trial (see Figure 1). Each condition was repeated 10 times for a total of 110 randomly placed trials. Before the experiment was executed, 10 random non-recorded trials were presented to train the participants. The duration of a single experiment was 10 min.

### 2.3. Statistical Analysis

Supposing a normal distribution of data (later confirmed by a Shapiro–Wilk test), quantitative variables were presented as mean ± standard deviation (SD). Qualitative data were presented as % frequency. For the statistical analysis, the SIFI results for children with migraine were compared through analyses of variance (ANOVA) to evaluate the variability inside a group (ANOVA with repeated measures) and between groups (ANOVA between). To evaluate the hypothesis that children with migraine show a reduction of illusion, we compared SIFIs in healthy children to children with migraine. 

## 3. Results 

We studied 26 migraine patients (14 males, 12 females) with an age of 11.30 ± 2.43 years. These were compared with 16 healthy subjects (8 males, 8 females) with an age of 10.61 ± 2.92 years. Among the female subjects, 7/12 (58.33%) patients and 3/8 (37.50%) healthy children had already started to have their period. Among the overall patients, the mean monthly migraine days were 4.31 ± 2.33, and the diagnosis had been performed from 34.42 ± 23.37 months; they did not show symptoms during the interictal period. Table 1 summarizes the patients’ clinical characteristics. 

### 3.1. Singe-Flash Trials

**Figure 2:**
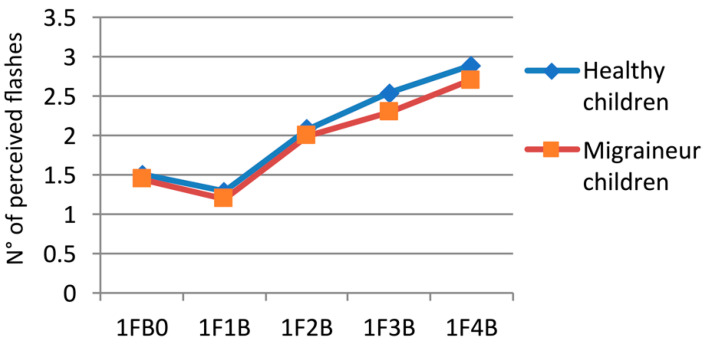
Fission illusions in children with migraine and healthy children during single-flash trials. On the x-axis, the flashes (F) and beeps (B) combination of the different trials are given: for example, 1FB0 means the presentation of one flash and no beep. On the y-axis, the mean number of perceived flashes is reported.

We performed an ANOVA with two factors: (i) conditions—single-flash trials with one flash and 0 to 4 beeps (five levels: 1F0B, 1F1B, 1F2B, 1F3B, 1F4B), and (ii) group (two levels: healthy subjects and migraineurs), which showed the following results (see Figure 2 and Table 2). In the interaction group for conditions: F (4, 160) = 0.42156, *p* = 0.79292; in the condition factor: F (4, 160) = 124.29, *p* = 0.00001; and in the group factor: F (1, 40) = 1.0179, *p* = 0.31907.

### 3.2. Multiple Flash Trials

**Figure 3:**
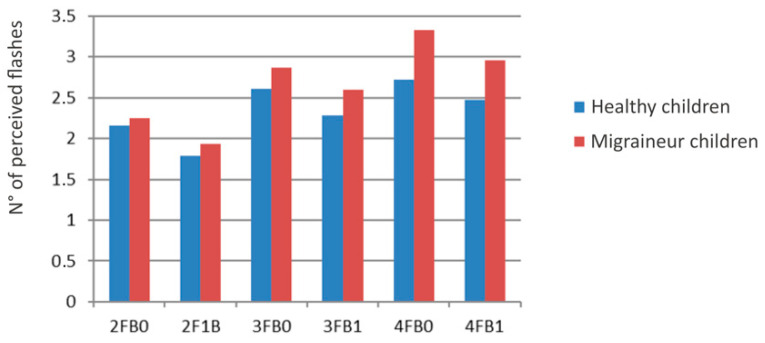
Fusion illusions in children with migraine and healthy children during multiple-flash trials. On the x-axis, the flashes (F) and beeps (B) combination of the different trials are given: for example, 1FB0 means the presentation of one flash and no beep. On the y-axis, the mean number of perceived flashes is reported.

**Figure 4:**
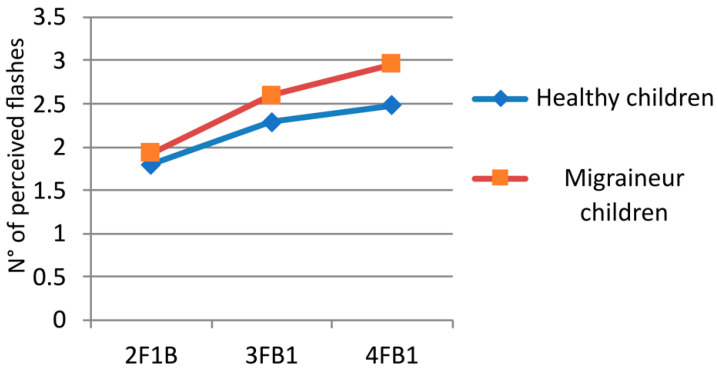
Flashes perceived with beeps during multiple-flash trials. On the x-axis, the flashes (F) and beeps (B) combination of the different trials are given: for example, 2F1B means the presentation of two flashes and one beep. On the y-axis, the mean number of perceived flashes is reported.

**Figure 5:**
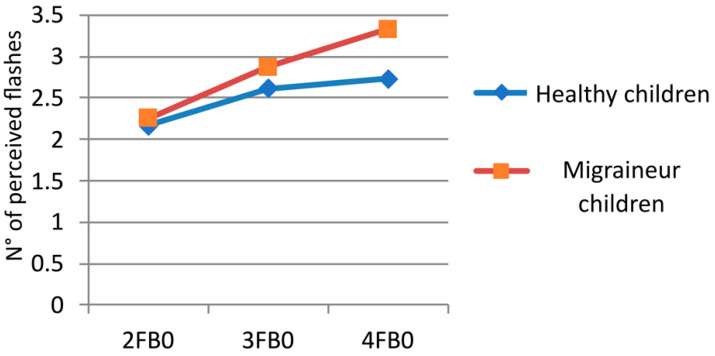
Flashes perceived without beeps during multiple-flash trials. On the x-axis, the flashes (F) and beeps (B) combination of the different trials are given: for example, 2FB0 means the presentation of two flashes and no beep. On the y-axis, the mean number of perceived flashes is reported.

We performed an ANOVA with three factors: (i) beep (two levels: 0 and 1), (ii) flash (three levels: 2-3-4), and (iii) group (two levels: healthy and migraineurs). The analysis showed the following results (Figure 3, Figure 4 and Figure 5 and Table 3): the interaction flash for groups showed a main effect of F (2, 80) = 12.280, *p* = 0.00002; the factor group showed F (1, 40) = 14.608, *p* = 0.00045; in the factor beep, we found F (1, 40) = 59.940, *p* = 0.00000; in the factor flash, there was F (2, 80) = 186.97, *p* = 0.00000; in the interaction beep for groups, F (1, 40) = 1.7348, *p* = 0.19530; in the interaction beep for flash, F (2, 80) = 0.44831, *p* = 0.64030; in the interaction beep for flash for groups, F (2, 80) = 0.32853, *p* = 0.72095. The flash interaction of the groups’ main effect is significant: F (2, 80) = 12.280, *p* = 0.00002. This indicates that the tendency to perceive a greater number of flashes in migraineurs increases with the number of flashes presented (without distinguishing between the presence or absence of beeps).

**Figure 6:**
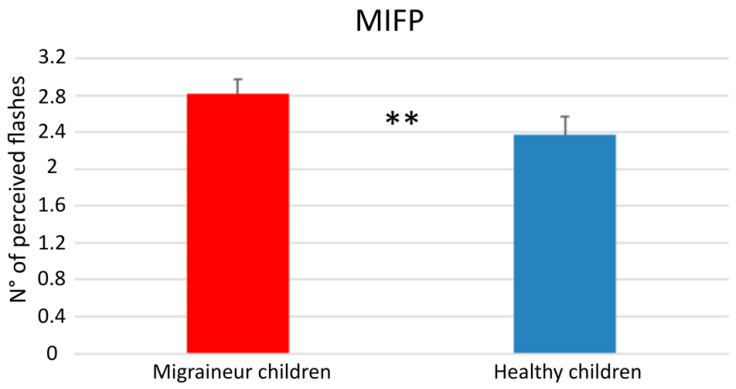
MIFP in children with migraine and healthy controls. MIFP: mean isolated flash perception. ** *p* < 0.01, using a *t*-test for unpaired data.

To highlight this difference, which represents the only significant difference between healthy children and migraineurs, we have also developed a measure for the perception of isolated flashes, expressed by the average value of flashes seen in all the conditions in which two or more flashes were presented without beeps (2F0B, 3F0B, 4F0B), which we defined as mean isolated flash perception (MIFP). The value of MIFP in migraineurs (2.89 ± 0.34) was significantly higher than in healthy children (2.31 ± 0.44), as determined using a t-test for unpaired data (*p* = 0.0052) (Figure 6).

## 4. Discussion

In this study, we explored SIFIs in children affected by migraine without aura to evaluate if changes like those observed in adults in cross-modal illusory audio-visual phenomena could be found also in the developmental age. This was to evaluate a condition called visual cortical excitability, as seen in adults, and to explore potential changes due to evolving connections regarding cross-modal interaction in this age range.

Results from single-flash trials (i.e., used to evaluate the fission illusions) showed a slight, but not significant, reduction in fission illusions among migraineurs compared to healthy children regarding trial 1F3B. Differently, in multiple-flash trials (i.e., designed to explore the fusion illusions), patients perceived significantly more flashes than healthy subjects. This result, however, could not be attributed to a reduced fusion illusions phenomenon as it occurred in the combined (i.e., flash and beeps) and isolated flash trials, as shown by the specific MIFP measure elaborated to evaluate trials containing only isolated flashes (see results).

SIFIs depend on the cross-modal interaction of the acoustic and visual cortex and are specifically related to the excitability levels of these structures. It is, in fact, possible to experimentally interfere with this illusory perception using pre-conditioning with anodal or cathodal tDCS and by increasing the degree of responsiveness of the occipital cortex or reducing that of the temporal cortex, as demonstrated in healthy subjects by Bolognini et al. (2011) [26]. These data seem to indicate that the acoustic stimuli drive illusory visual perception: when these are reduced due to the inhibition of the acoustic cortex, or the transmission is less effective due to the increase in excitability of the visual cortex, the illusion disappears or fades. Brighina et al. observed a reduction in the illusory phenomenon in migraine patients that occurs both in the interictal phase (in migraineurs with aura) and during the attack (in patients with and without aura) [27]. This evidence fits well with the hypothesis of visual cortical hyperexcitability in migraine, which is present not only in the interictal condition (especially in patients with aura) but also during the attack when the lowest levels of fission illusions (i.e., highest cortical excitability) are reached. The result obtained by Brighina et al. (2015) underlines the importance of visual cortical hyperexcitability both in migraine with aura and without aura [27]. So, if hyperexcitability can represent the basis for triggering CSD in migraine with aura, it could well play a pathophysiological role in migraine without aura. This strengthens the hypothesis that, even in migraine with aura, a CSD-like mechanism called non-symptomatic CSD involving only silent cortical areas, even if not producing clinically appreciable aura phenomena, could still be capable of activating the trigeminal vascular system and triggering headache symptoms [32].

Neurophysiological investigations with different techniques have provided evidence about increased cortical excitability in adult migraineurs, underlining the importance not just of functional connectivity of cortical areas in the precipitation of the attack. Studies with TMS have shown an increase in cortical excitability (assessed by PT) in basic conditions or as a paradoxical response after rTMS at 1 Hz on the visual cortex compared to controls. An increase in excitability was also observed for visual associative areas responsible, for example, for the perception of moving images (i.e., V5 or motion-sensitive area MT), observing how the threshold for the induction of moving phosphenes—typically evoked by the stimulation of these areas—is reduced in migraineurs. Data in favor of greater cortical activation also come from studies performed with less subjective techniques compared to phosphenes, such as the magnetic suppression of perceptual accuracy (MPSA), which evaluates the interference induced by occipital magnetic stimulation on visual perceptual accuracy (percentage of recognition of groups of letters presented on a screen during occipital TMS) [33]. It is more difficult to generate interference effects on visual perception with occipital TMS in patients suffering from migraine with aura, and this effect is attributed to the lower efficiency of the inhibitory circuits [34,35].

Neurophysiological studies with visually evoked potentials conducted in migraineurs of developmental age have shown a greater amplitude of responses after visual stimulus compared to healthy subjects, confirming the abnormalities already found in adult migraineurs. Relevant insights regarding children with migraine also came about through the study of the recovery cycle of sensory-evoked potentials (i.e., a marker of cortical inhibitory efficiency) applied to evaluate the excitability of the somatosensory cortex. The results showed that the amplitude of the recovery cycle of cervical N13, N20, and P24 and cortical N30 was reduced compared to healthy controls, supporting the lower efficiency of cortical inhibition in migraineurs during childhood [36,37]. 

One of the objectives of our study was to investigate whether the hyperexcitability in migraine could modulate illusory perceptions in an immature multisensory integration system. The migraineurs’ perception of fission illusions showed to be at the same level as healthy subjects, but from Figure 2, we can observe for the 1F3B combination, a minimal, albeit not significant, difference. Such results could suggest that the hyperexcitability of the occipital cortex requires a long time before it can emerge as it does in adult migraineurs because, when an individual is at an evolutionary age, the auditory dominance associated with multiple beeps interferes more with the determinism of the fission illusion. This indeed supports the data according to which the maturation of audio-visual integration systems occurs in conjunction with schooling [38]. As demonstrated by Nava et al. (2013), through sound-induced flash illusions, during growth, a transition occurs from auditory to visual sensory dominance in cross-modal perception processes [29]. 

However, it is not to be ruled out that the absence of a reduced number of fission illusions is due to the lack of opportunity to test subjects during the attack when greater visual cortical excitability is assumed [27]. In support of greater cortical excitability during an attack, Xiang et al. [37] demonstrated the presence of a dysfunction of the excitability of the motor cortex in migraine-suffering children through magnetoencephalography (MEG), where cerebral activation was elicited by finger tapping. The results showed a very high rate of activation of the cortex during the ictal phase and normalization during the interictal phase [38]. Other studies conducted with MEG and fMRI have confirmed that by inducing visual stress in patients with migraine, anomalous excitability of various cortical areas, such as the occipital, occipital-temporal, and occipital-parietal areas, is established, which is capable of triggering the aura or the attack, respectively, in migraineurs with or without aura [39,40].

However, from the analysis of multiple-flash trials, it emerged that even if migraineurs of developmental age do not perceive fewer illusions, they are able to perceive and discriminate multiple flashes better than controls of the same age (*p* = 0.00002). These data point out how children with migraine show an increased visual discrimination capacity even outside the attack. The visual system is relevant in migraine, as demonstrated for example by the symptom of photophobia, or by the role of cortical excitability and cortical activation (i.e., CSD) in aura determinism and migraine attacks. Further confirmations come from the neurophysiological studies with visual evoked potentials in children with migraine that showed a greater response after visual stimulation compared to healthy subjects [41]. Studies using TMS in the occipital cortex (with PT and suppression of visual perception) in migraine children without aura showed that migraineurs presented lower PTs than healthy participants at each time point, indicating increased occipital excitability. This was attenuated 1–2 days before a migraine attack, as indicated by a relative increase in PT. However, the increase in PTs before the next attack was associated with a stronger TMS-induced suppression of visual perception and a prolongation of the motion aftereffect. These findings show that pediatric migraine without aura is associated with a systematic shift in occipital excitability preceding the migraine attack [41,42,43].

Further suggestions come from this study, as by non-invasively studying the cortical excitability of children, it could be possible to monitor the efficacy of preventive therapies in patients with high-frequency migraine attacks. Supporting evidence comes from the study by Vollono et al. [44] in which improvement in the recovery cycle of somatosensory-evoked potential components with the use of topiramate was accompanied by an improvement in the frequency of migraine attacks. Conversely, subjects in whom this restoration of the cycle did not occur showed an ineffective response to topiramate. The authors concluded that their results suggest that topiramate efficacy was probably related to restored cortical excitability. The possibility of studying the efficacy of different pharmacological and non-pharmacological treatments in children at an early stage is also useful given parents’ fear of the side effects of therapy. Further, cortical excitability in children with migraine seems to correlate with behavioral symptomatology [45].

In conclusion, we affirm the importance of multiple-flash trials to evaluate visual function in migraine during childhood. Even at an evolutionary age, there is already a remarkable hyper-responsiveness of the visual cortex, allowing us to say that probably the alterations of cortical excitability are already present at this age. Thus, the present study adds to the literature insight about a non-invasive marker of visual cortical function even in children with migraine.

This study, however, is still to be considered preliminary, and the following limitations are to be mentioned: it is necessary to conduct further experiments in the ictal phase to evaluate whether, similar to that which occurs in adults, the visual cortical excitability increases to oppose the illusory effect of acoustic stimulation during the audio-visual illusions. It is equally important to evaluate migraine children with aura in which a greater cortical excitability has been shown, or in pediatric chronic migraine where a more elevated cortical excitability was demonstrated compared to episodic migraine. Furthermore, the limited number of samples does not allow us to evaluate our results as a function of age, sex, frequency of attacks, and duration, which, in light of the above-mentioned considerations on the maturation process in children and variations in phenotype during developmental ages [2,3], are aspects that should not be overlooked. The absence in the control population of subjects suffering from tension-type headaches is an aspect that will need to be examined because at a developmental age, the boundaries between the two disorders are less clear than in adulthood, and there is frequently a transition from one form of headache to the other (more frequently from a tension-type headache to migraine) [46,47]. Finally, it would also be appropriate to compare our results with other non-invasive methods such as evoked potentials, quantitative EEG, etc., in the same subjects [48].

However, we believe that this study can help generate new hypotheses for research using non-invasive methods, and clinically, supported by other studies, SIFIs could represent an early marker of cortex hyper-responsiveness to identify pediatric migraine. 

## Figures and Tables

**Table 1:** Migraine population clinical characteristics.

Variable	Mean	SD
Migraine frequency (days/month)	4.31	2.33
Months since diagnosis	34.42	23.37
Headache intensity (NRS)	7.50	1.45
Attack duration (hours)	5.96	6.31

Abbreviations: NRS = numeric rating scale; SD = standard deviation.

**Table 2:** Single-flash trial results.

Test	Group	Mean	SD
1FB0	HC	1.51	0.396
	MC	1.44	0.403
1F1B	HC	1.29	0.403
	MC	1.20	0.251
1F2B	HC	2.08	0.650
	MC	1.99	0.412
1F3B	HC	2.54	0.595
	MC	2.30	0.603
1F4B	HC	2.89	0.673
	MC	2.71	0.725

Abbreviations: HC = healthy children; MC = migraineur children; SD = standard deviation.

**Table 3:** Multiple-flash trial results.

Test	Group	Mean	SD
2FB0	HC	2.06	0.447
	MC	2.25	0.380
2F1B	HC	1.57	0.562
	MC	1.93	0.403
3FB0	HC	2.38	0.470
	MC	2.87	0.407
3FB1	HC	1.95	0.670
	MC	2.60	0.465
4FB0	HC	2.67	0.575
	MC	3.33	0.429
4FB1	HC	2.23	0.662
	MC	2.96	0.522

Abbreviations: HC = healthy children; MC = migraineur children; SD = standard deviation.

## Data Availability

Data are available upon reasonable request to the corresponding author. The data are not publicly available due to privacy restrictions.

## References

[1] Onofri A., Pensato U., Rosignoli C., Wells-Gatnik W., Stanyer E., Ornello R., Chen H.Z., De Santis F., Torrente A., Mikulenka P. (2023). Primary headache epidemiology in children and adolescents: A systematic review and meta-analysis. J. Headache Pain.

[2] Lewis D.W. (2007). Pediatric migraine. Pediatr. Rev..

[3] Raieli V., D’Amico A., Piro E. (2020). Migraine in Children Under 7 Years of Age: A Review. Curr. Pain Headache Rep..

[4] García-Azorín D., Moya-Alarcón C., Armada B., Sánchez del Río M. (2024). Societal and economic burden of migraine in Spain: Results from the 2020 National Health and Wellness Survey. J. Headache Pain.

[5] Mazzotta G., Gallai B., Mattioni A., Floridi F., Foti F., Allegretti M., D’angelo R. (2005). Cost assessment of headache in childhood and adolescence: Preliminary data. J. Headache Pain.

[6] Finning K., Neochoriti Varvarrigou I., Ford T., Panagi L., Ukoumunne O.C. (2022). Mental health and school absenteeism in children with long-term physical conditions: A secondary analysis of the British Child and Adolescent Mental Health Surveys 2004 and 2007. Child Care Health Dev..

[7] Dodick D.W. (2018). A Phase-by-Phase Review of Migraine Pathophysiology. Headache.

[8] Olesen J. (2018). Headache Classification Committee of the International Headache Society (IHS) The International Classification of Headache Disorders, 3rd edition. Cephalalgia.

[9] Bose P., Karsan N., Goadsby P.J. (2018). The Migraine Postdrome. Contin. Lifelong Learn. Neurol..

[10] Gelfand A.A., Goadsby P.J., Allen I.E. (2015). The relationship between migraine and infant colic: A systematic review and meta-analysis. Cephalalgia.

[11] Özge A., Abu-Arafeh I., Gelfand A.A., Goadsby P.J., Cuvellier J.C., Valeriani M., Sergeev A., Barlow K., Uludüz D., Yalın O. (2017). Experts’ opinion about the pediatric secondary headaches diagnostic criteria of the ICHD-3 beta. J. Headache Pain.

[12] Raieli V., Capizzi M., Marino A., Di Nardo G., Raucci U., Parisi P. (2022). Study on “Atypical” Migraine Auras in the Pediatric Age: The Role of Cortical Spreading Depression and the Physiopathogenetic Hypothesis Arising from Our Clinical Cases. Life.

[13] Gelfand A.A., Reider A.C., Goadsby P.J. (2013). Cranial autonomic symptoms in pediatric migraine are the rule, not the exception. Neurology.

[14] Jernigan T.L., Baaré W.F.C., Stiles J., Madsen K.S. (2011). Postnatal brain development: Structural imaging of dynamic neurodevelopmental processes. Prog. Brain Res..

[15] Stiles J., Jernigan T.L. (2010). The basics of brain development. Neuropsychol. Rev..

[16] Guidetti V., Galli F., Termine C. (2010). Headache in children. Handb. Clin. Neurol..

[17] Deodato M., Granato A., Martini M., Stella A.B., Galmonte A., Murena L., Manganotti P. (2024). Neurophysiological and Clinical Outcomes in Episodic Migraine without Aura: A Cross-Sectional Study. J. Clin. Neurophysiol..

[18] Goadsby P.J., Holland P.R., Martins-Oliveira M., Hoffmann J., Schankin C., Akerman S. (2017). Pathophysiology of Migraine: A Disorder of Sensory Processing. Physiol. Rev..

[19] Dichgans M., Freilinger T., Eckstein G., Babini E., Lorenz-Depiereux B., Biskup S., Ferrari M.D., Herzog J., van den Maagdenberg A.M.J.M., Pusch M. (2005). Mutation in the neuronal voltage-gated sodium channel SCN1A in familial hemiplegic migraine. Lancet.

[20] Van den Maagdenberg A.M., Pietrobon D., Pizzorusso T., Kaja S., Broos L.A., Cesetti T., van de Ven R.C., Tottene A., van der Kaa J., Plomp J.J. (2004). A Cacna1a knockin migraine mouse model with increased susceptibility to cortical spreading depression. Neuron.

[21] Ophoff R.A., Terwindt G.M., Vergouwe M.N., van Eijk R., Oefner P.J., Hoffman S.M., Lamerdin J.E., Mohrenweiser H.W., Bulman D.E., Ferrari M. (1996). Familial hemiplegic migraine and episodic ataxia type-2 are caused by mutations in the Ca^2+^ channel gene CACNL1A4. Cell.

[22] Creutzfeldt O.D., Fromm G.H., Kapp H. (1962). Influence of transcortical d-c currents on cortical neuronal activity. Exp. Neurol..

[23] Cosentino G., Di Marco S., Ferlisi S., Valentino F., Capitano W.M., Fierro B., Brighina F. (2018). Intracortical facilitation within the migraine motor cortex depends on the stimulation intensity. A paired-pulse TMS study. J. Headache Pain.

[24] Brighina F., Piazza A., Daniele O., Fierro B. (2002). Modulation of visual cortical excitability in migraine with aura: Effects of 1 Hz repetitive transcranial magnetic stimulation. Exp. Brain Res..

[25] Shams L., Kamitani Y., Shimojo S. (2000). What you see is what you hear. Nature.

[26] Bolognini N., Rossetti A., Casati C., Mancini F., Vallar G. (2011). Neuromodulation of multisensory perception: A tDCS study of the sound-induced flash illusion. Neuropsychologia.

[27] Brighina F., Bolognini N., Cosentino G., Maccora S., Paladino P., Baschi R., Vallar G., Fierro B. (2015). Visual cortex hyperexcitability in migraine in response to sound-induced flash illusions. Neurology.

[28] Innes-Brown H., Barutchu A., Shivdasani M.N., Crewther D.P., Grayden D.B., Paolini A.G. (2011). Susceptibility to the flash-beep illusion is increased in children compared to adults. Dev. Sci..

[29] Nava E., Pavani F. (2013). Changes in sensory dominance during childhood: Converging evidence from the colavita effect and the sound-induced flash illusion. Child. Dev..

[30] Afra J., Mascia A., Gérard P., Maertens de Noordhout A., Schoenen J. (1998). Interictal cortical excitability in migraine: A study using transcranial magnetic stimulation of motor and visual cortices. Ann. Neurol..

[31] Headache Classification Committee of the International Headache Society (IHS) (2013). The International Classification of Headache Disorders, 3rd edition (beta version). Cephalalgia.

[32] Zhang X., Levy D., Kainz V., Noseda R., Jakubowski M., Burstein R. (2011). Activation of central trigeminovascular neurons by cortical spreading depression. Ann. Neurol..

[33] Mulleners W.M., Chronicle E.P., Palmer J.E., Koehler P.J., Vredeveld J.W. (2001). Visual cortex excitability in migraine with and without aura. Headache.

[34] Battelli L., Black K.R., Wray S.H. (2002). Transcranial magnetic stimulation of visual area V5 in migraine. Neurology.

[35] Custers A., Mulleners W.M., Chronicle E.P. (2005). Assessing cortical excitability in migraine: Reliability of magnetic suppression of perceptual accuracy technique over time. Headache.

[36] Pro S., Tarantino S., Capuano A., Vigevano F., Valeriani M. (2014). Primary headache pathophysiology in children: The contribution of clinical neurophysiology. Clin. Neurophysiol..

[37] Xiang J., Degrauw X., Korostenskaja M., Korman A.M., O’Brien H.L., Kabbouche M.A., Powers S.W., Hershey A.D. (2013). Altered cortical activation in adolescents with acute migraine: A magnetoencephalography study. J. Pain.

[38] Leiken K.A., Xiang J., Curry E., Fujiwara H., Rose D.F., Allen J.R., Kacperski J.E., O’brien H.L., Kabbouche M.A., Powers S.W. (2016). Quantitative neuromagnetic signatures of aberrant cortical excitability in pediatric chronic migraine. J. Headache Pain.

[39] Bowyer S.M., Aurora K.S., Moran J.E., Tepley N., Welch K.M. (2001). Magnetoencephalographic fields from patients with spontaneous and induced migraine aura. Ann. Neurol..

[40] Cao Y., Welch K.M., Aurora S., Vikingstad E.M. (1999). Functional MRI-BOLD of visually triggered headache in patients with migraine. Arch. Neurol..

[41] Akin O., Ünay B., Arslan M., Mazman S., Taşçilar E., Eker I. (2018). Evaluation of somatosensory evoked potentials (SEPs) in obese children. Gulhane Med. J..

[42] Ernst M.O. (2008). Multisensory integration: A late bloomer. Curr. Biol..

[43] Siniatchkin M., Reich A.L., Shepherd A.J., van Baalen A., Siebner H.R., Stephani U. (2009). Peri-ictal changes of cortical excitability in children suffering from migraine without aura. Pain.

[44] Vollono C., Ferraro D., Miliucci R., Vigevano F., Valeriani M. (2010). The abnormal recovery cycle of somatosensory evoked potential components in children with migraine can be reversed by topiramate. Cephalalgia.

[45] Valeriani M., Galli F., Tarantino S., Graceffa D., Pignata E., Miliucci R., Biondi G., Tozzi A., Vigevano F., Guidetti V. (2009). Correlation between abnormal brain excitability and emotional symptomatology in paediatric migraine. Cephalalgia.

[46] Baglioni V., Orecchio S., Esposito D., Faedda N., Natalucci G., Guidetti V. (2023). Tension-Type Headache in Children and Adolescents. Life.

[47] Onan D., Younis S., Wellsgatnik W.D., Farham F., Andruškevičius S., Abashidze A., Jusupova A., Romanenko Y., Grosu O., Moldokulova M.Z. (2023). Debate: Differences and similarities between tension-type headache and migraine. J. Headache Pain.

[48] Puca F., de Tommaso M. (1999). Clinical neurophysiology in childhood headache. Cephalalgia.

